# A Case Series Highlighting the Relative Frequencies of the Common, Uncommon and Atypical/Unusual Hematological Findings on Bone Marrow Examination in Cases of Visceral Leishmaniasis

**DOI:** 10.4084/MJHID.2011.035

**Published:** 2011-09-08

**Authors:** Prateek Bhatia, Deepanjan Haldar, Neelam Varma, RK Marwaha, Subhash Varma

**Affiliations:** 1 Department of Hematology; 2 Department of Pediatrics; 3 Department of Internal Medicine. Institute of Medical Education and Research, Chandigarh. India

## Abstract

**Introduction:**

Bone marrow aspiration and biopsy still remains as one of the vital tests for confirmation of diagnosis of visceral Leishmaniasis. The aim of the present study is to assess the relative frequency of common, uncommon and atypical hematological findings in cases of Visceral Leishmaniasis.

**Materials & Methods:**

A total of 16 cases of Leishmaniasis diagnosed on Bone marrow examination over a period of two years (2008–2010), were retrieved from the archives and the peripheral blood smear, bone marrow aspiration smears and trephine biopsies were examined for the common, uncommon and atypical features as described in the literature.

**Results:**

Out of the total of 16 cases, 10 were pediatric and 6 adult cases. The common findings like pancytopenia, peripheral blood monocytosis, increased histiocytes on aspirate smears and granulomas on biopsies were noted in 12/16 (75%), 9/16 (56.25%), 13/16 (81.2%) and 11/16 (69%) cases respectively. Amongst the uncommon findings, hemophagocytosis was noted in 12/16 (75%) cases, plasma cells with inclusions in 6/16 (37.5%) and LD bodies in cells other than histiocytes in 4/16 (25%) cases. The atypical findings included organism aggregates noted in 9/16 (56%) cases, Pelger-Heut cells seen in 4/16 (25%) cases and increased focal vascularity on biopsies in 10/16 (62.5%) cases. The average parasite density (APD) on smears was 3+ and the range of positivity was 1+ to 5+.

**Conclusion:**

The knowledge of these morphological clues can assist us in searching for LD bodies and correctly diagnosing the condition without excessive dependence on unnecessary and sophisticated tests.

## Introduction:

Visceral Leishmaniasis has a high prevalence in India with endemicity in many regions. It usually presents clinically with derangement of hematological parameters. Bone marrow aspiration and biopsy remains as one of the vital tests for confirmation of diagnosis. Though there are many reports on bone marrow findings in Leishmaniasis, but only a limited few highlight the importance of uncommon and atypical morphological features helpful in diagnosing Leishmaniasis on aspirate smears. The aim of the present study is to assess the relative frequencies of these morphological findings on marrow aspirate and biopsies and their importance in early disease diagnosis.

## Materials and Methods:

A total of 16 cases of Leishmaniasis diagnosed on Bone marrow examination over a period of two years (2008–2010), were retrieved from the archives and the peripheral blood smear, bone marrow aspiration and biopsies were examined for various common, uncommon and atypical findings. The Geimsa stained slides of bone marrow aspirates and hematoxylin and Eosin stained biopsy slides were reviewed and discussed in detail by three competent hemato-pathologists. All the findings were tabulated, discussed and deliberated upon and then graded as common, uncommon and atypical findings based on a previous study by Yahya Daneshbod et al.^1^ The common findings included-Peripheral blood pancytopenia, monocytosis and nRBC’s, Bone marrow aspirate-Average Parasite Density, plasmacytosis, reversed M:E ratio with dyserythropoiesis, histiocytes and LD body distribution and on Trephine biopsies-the presence of granulomas. The uncommon findings included-presence of LD bodies in cells other than histiocytes, plasma cells with abnormal inclusions, presence of blasts/hematogones and the grade of hemophagocytosis. The atypical/unusual findings include-pseudo Pelger-Heut cells, increased vascularity and fibrosis on biopsies, atypical histiocytes morphology and aggregates of LD bodies. The Following parameters were graded on aspirate smears according to the standard grading protocols described in literature: Average Parasite Density (APD) graded on smears^2^ as (0)- 0 LD Bodies/1000 Fields, (1)+- 1-10 LD Bodies/1000 Fields, (2+)- 1-10 LD Bodies/100 Fields, (3+)- 1-10 LD Bodies/10 Fields, (4+)- 1-10 LD Bodies/Field, (5+)- 10-100 LD Bodies/Field and (6+)- >100 LD Bodies/Field. Hemophagocytosis (HPS) was graded on aspirate smears^3^ as- (0)-Absent; (1+) (Mild)- <2 histiocytes with HPS /Slide; (2+) (Moderate)-2–5 histiocytes with HPS/Slide; (3+) (Severe)->5 histiocytes with HPS/Slide. Vascularity was graded on trephine biopsies^4^ (H&E; Visual) as-(Focal)-Increased visual density of vessels on H&E/Marrow space but involving< 50% of the Biopsy and (Diffuse)-Increased visual density of vessels on H&E/Marrow space but involving> 50% of the Biopsy.

## Results:

**Figure 1.**
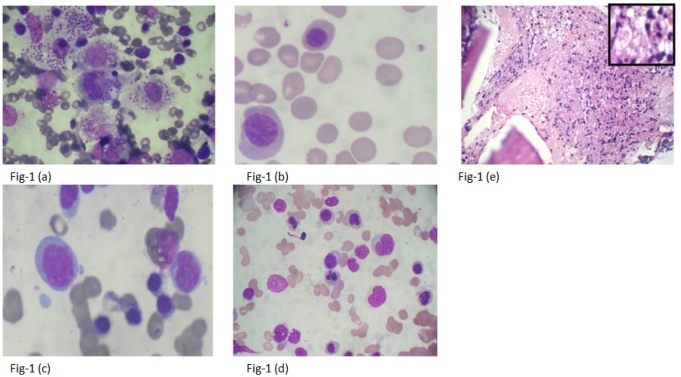
Common Findings in cases of Visceral Leishmaniasis. 1 (a): Intrahistiocytic LD bodies- APD5+(1000x). 1 (b): Free cytoplasmic LD bodies-APD2+ (1000x). 1 (c): Erythroid hyperplasia with moderate megaloblastosis(1000x). 1 (d): Dyserythropoiesis (1000x). 1 (e): An Ill formed Paratrabecular Granuloma showing presence of epithelioid cells and mixed inflammatory infiltrate (H&E,400x); Inset LD bodies within epithelioid cells(1000x)

**Figure 2.**
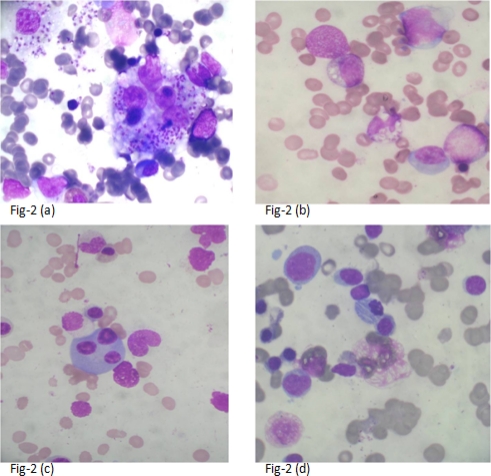
Uncommon findings in cases of Visceral Leishmaniasis. 2 (a): Intrahistiocytic LD bodies & Hemophagocytosis (1000x). 2 (b): LD bodies inside neutrophil (1000x). 2 (c): Trinucleated plasma cell (1000x). 2 (d): Plasma cells with abnormal crystalline inclusions of immunoglobulin’s (1000x)

Out of the total of 16 cases, 10 were pediatric cases and 6 adult cases. The relative frequencies of each of the common, uncommon and atypical findings are highlighted in Tables 1, 2 **and** 3 respectively. The various morphological findings are also shown in figures 1(a–e), 2 (a–d) **and** 3 (a–e).

## Discussion:

Visceral Leishmaniasis can affect wide age group of patients. In this series the age ranged from 2 months to 65 years with three patients being under one year of age in none of whom visceral Leishmaniasis was suspected clinically. In these infants the average parasite density on smears ranged from 1+-2+ and the uncommon and atypical findings were all present except for increased fibrosis and blasts/hematogones, which were seen in only one of the infant. This highlights the fact that the presence of uncommon or atypical findings should prompt a diligent search for LD bodies even if the cases are asymptomatic clinically.

Splenic sequestration, ineffective hematopoiesis and hemophagocytosis appear to be the main etiopathogenetic factors in the emergence of hypercellular marrow with peripheral cytopenias. Reversal in myeloid erythroid ratio associated with megaloblastosis and mild to moderate dyserythropoiesis is also quiet common in Leishmaniasis and well documented in literature.^5^ Additional treatment with Vitamin B 12 and folic acid in these patients have been shown to improve the anemia faster.

Presence of granulomas in patients with pyrexia of unknown origin should also prompt a search of LD bodies and should not be considered synonymous with tuberculosis. In fact necrotizing granulomas in patients with visceral Leishmaniasis have been shown to be associated with poor prognosis.^6^ Hemophagocytosis although classified as an uncommon finding previously, 7 has been observed much more commonly in our patients and in fact was the most common morphological finding followed by presence of granulomas and plasmacytosis. This could be due to longer duration of symptoms in patients before seeking medical attention and delayed referral to our hospital from far-off endemic regions. Visceral Leishmaniasis must be considered and excluded in patients with hemophagocytosis before immunosuppressive therapy is considered.

Increased vessel density was also a common finding, though it was predominantly focal in 9 cases and diffuse in only one case. This increased vascularity could be because of release of cytokines secondary to infection leading on to neoangiogenesis.

Aggregates of LD bodies are also described in literature and these aggregates can be flower like, clover-leaf shaped, ball like or irregular star shaped. It is important to recognize them and be aware of them so that they are not confused with platelet aggregates. The presence of these aggregates extracellularly in cases of Leishmaniasis is a common finding as highlighted in our series wherein they were noted in 9/16 (57%) cases.

## Conclusion:

Knowledge of common, uncommon and atypical /unusual findings of visceral Leishmaniasis would prompt more diligent search of the organism and thus help in arriving at a correct diagnosis. Presence of certain findings such as associated hemophagocytosis and necrotizing granulomas should be communicated to the treating clinician and might be helpful in planning further management.

## Figures and Tables

**Figure 3.**
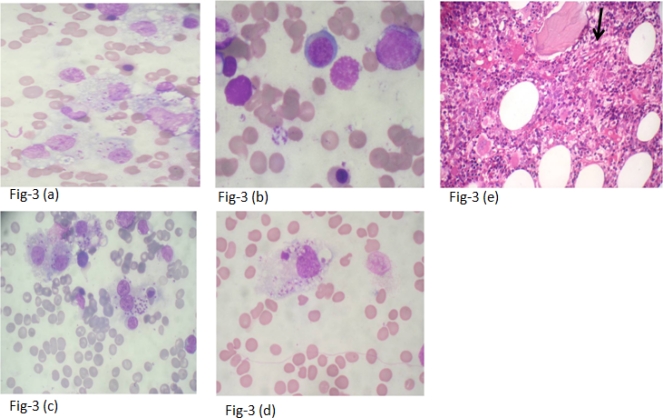
Atypical findings in cases of Visceral Leishmaniasis. 3 (a): Sheets of foamy histiocytes containing LD bodies (1000x). 3 (b): Flower like aggregate of LD bodies (1000x). 3 (c): Histiocytes with LD bodies (RS like cell) (1000x). 3 (d): Tart cell – a histiocytes containing cellular debris with LD bodies (1000x). 3 (e): Increased vessel density (arrow;H&E,400x)

**Table 1.** Common findings in bone marrow aspirates from patients with Visceral Leishmaniasis.

	FINDINGS		CASES(%){n=16}
Peripheral Blood Smear	Pancytopenia		12(75)
	Monocytosis		10(62)
	n RBC’s		11(69)
Bone Marrow Aspirate	Plasmacytosis		9(56)
	Reverse M:E Ratio with Dyserythropoiesis		6(38)
	Increased Histiocytes		13(81)
	Distribution of LD bodies	Intra Histiocytic	7(44)
		Extra Histiocytic	5(31)
		Equal	4(25)
Trephine biopsy	Granulomas	Non Necrotizing	9(56)
		Necrotizing	2(12)
Aspirate Smear	Average Parasite Density	(APD)	1+ (2 cases); 2+ (5 cases); 3+ (6 cases); 4+ (1 case); 5+ (2 cases)

**Table 2.** Uncommon findings in aspirates from patients with visceral Leishmaniasis.

FINDINGS		CASES(%){n=16}
Presence of Organisms in cells other than Histiocytes		Polymorhs -3(19) Metamyelocytes -1(6)
Plasma cells with abnormal inclusions		6(38)
Increased blasts/hematogones		1(6)
***Hemophagocytosis***	Mild	3(19)
	Moderate	5(31)
	Severe	4(25)

**Table 3.** Atypical findings in aspirates from patients with visceral Leishmaniasis

FINDINGS		CASES(%){n=16}
Pseudo -Pelger Huet		4(25)
***Increased vascularity***		10(63)
Increased fibrotic foci		3(19)
Atypical histiocytic morphology (Tart cell, RS like, foam cells etc.)		4(25)
Aggregates Of LD bodies	Regular	7(44)
	Irregular	2(13)
